# A structure–kinetic relationship study using matched molecular pair analysis†

**DOI:** 10.1039/d0md00178c

**Published:** 2020-09-21

**Authors:** Doris A. Schuetz, Lars Richter, Riccardo Martini, Gerhard F. Ecker

**Affiliations:** Department of Pharmaceutical Chemistry, University of Vienna UZA 2, Althanstrasse 14 1090 Vienna Austria gerhard.f.ecker@univie.ac.at

## Abstract

The lifetime of a binary drug–target complex is increasingly acknowledged as an important parameter for drug efficacy and safety. With a better understanding of binding kinetics and better knowledge about kinetic parameter optimization, intentionally induced prolongation of the drug–target residence time through structural changes of the ligand could become feasible. In this study we assembled datasets from 21 publications and the K4DD (Kinetic for Drug Discovery) database to conduct large scale data analysis. This resulted in 3812 small molecules annotated to 78 different targets from five protein classes (GPCRs: 273, kinases: 3238, other enzymes: 240, HSPs: 160, ion channels: 45). Performing matched molecular pair (MMP) analysis to further investigate the structure–kinetic relationship (SKR) in this data collection allowed us to identify a fundamental contribution of a ligand's polarity to its association rate, and in selected cases, also to its dissociation rate. However, we furthermore observed that the destabilization of the transition state introduced by increased polarity is often accompanied by simultaneous destabilization of the ground state resulting in an unaffected or even worsened residence time. Supported by a set of case studies, we provide concepts on how to alter ligands in ways to trigger on-rates, off-rates, or both.

## Abstract 2

A large-scale study employing matched molecular pair (MMP) analysis to uncover the contribution of a compound's polarity to its association and dissociation rates.

## Graphical abstract

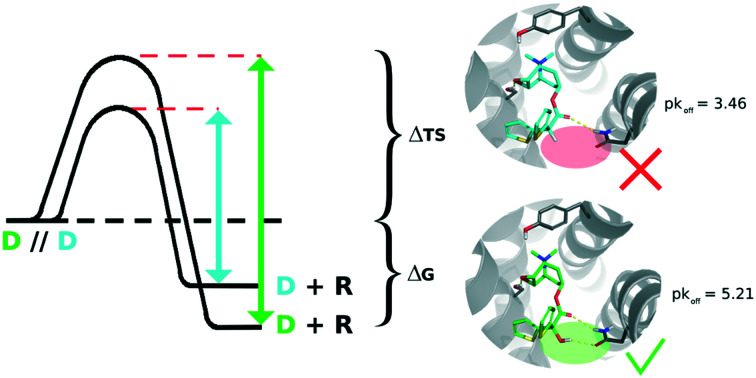

## Introduction

### Importance of kinetic parameters in drug design

Multiple studies on the kinetic behavior of small molecules show how the lifetime of a binary drug–target complex is inevitable for translation into *in vivo* efficacy.^1–6^ The so-called drug residence time (*τ*), which is the time a drug spends bound to its protein target, not only influences efficacy, but is also linked to toxicity^7^ and off-target activity.^8,9^ The life span of this complex does not only need to be of minimal duration to achieve a certain function, but also, in particular cases, should not exceed a certain time for optimal function.^10^ Therefore, the residence time of a drug might be a key determinant for clinical success of drug candidates.^11^

The two important kinetic parameters in drug–target binding kinetics are the on-rate and the off-rate. The on-rate or association rate, *k*_on_, is a measure of how fast a molecule binds to its biological target. The off-rate, *k*_off_, is the dissociation rate, which is the parameter most scientific publications have focused on. It is the inverse of the residence time, and therefore a measure for how long a compound remains bound to its protein target. The off-rate *k*_off_ can be influenced in 2 ways: i) stabilization of the ground state^12^ and/or ii) destabilization of the transition state.^13–15^ In both cases, the energy difference between the bound state and the transition state needs to be increased to reach higher *τ* values. Stabilization of the ground state translates into improved affinity of the drug towards its target, which has been widely studied. In contrast, achieving an increase of residence time by destabilizing the transition state is a less explored field. There are only a few examples in the literature highlighting how to impact the transition state.^14–16^ In particular, it has been reported that different drugs with the same affinity for a given protein exhibit totally different kinetic behaviours.^13^ While the difference in energy between the bound state and the unbound state refers to the affinity, the difference in energy between the unbound state and the transition state can be directly translated into the association constant (*k*_on_). Increasing the energy barrier to overcome the transition state results in a slower binding event (the *k*_on_ value gets smaller), and consequently in a prolonged residence time (assuming two different molecules display similar binding affinities) and the system is in an equilibrium state (where *K*_D_ = *k*_off_/*k*_on_). Therefore, to understand how to trigger the residence time it is of crucial importance to grasp the relationship between structural modifications of a molecule and the effect on its *k*_off_ and *k*_on_ profile. While the functional efficacy is often correlated to the residence time,^3^ Copeland observed that *k*_on_ also has to be considered for the pharmacological action of a drug.^17^ It contributes to kinetic selectivity by displaying different binding pathways for yet identical binding pockets.^13^ Furthermore, it translates into cellular and *in vitro* effects.^18^ Apart from that, the on-kinetics of a ligand significantly affect its profile and side effects, which has recently been demonstrated for the dopamine D2 receptor.^19^ Additionally, on-rates have been shown to be of significant importance for target occupancy^20,21^ and contribute to drug rebinding.^22^ It has also been shown that on-rates translate into kinetically biased agonism towards different pathways. Thus, Herenbrink *et al.* published that on-rates are the determining factor in GPCR downstream pathway prioritization, leading to different biological outcomes.^23^

In order to systematically explore the effect of distinct structural modifications on the kinetic profile of compound–target associations, we derived the hitherto largest kinetic dataset (KIND) available in the literature and used the kinetic data triplets for extensive matched molecular pair analysis.

## Results and discussion

### KIND (KINetic Dataset)

The kinetic dataset KIND (KINetic Dataset) contains a total of 3812 structures and their kinetic data triplets (*k*_on_, *k*_off_, *K*_D_). It has been compiled from 21 publications^16,19,24–42^ and the K4DD database (for details see the ESI,† the dataset is provided in KIND.xlsx). For the literature search, only papers containing numerical values for all three parameters investigated (*K*_D_, *k*_on_ and *k*_off_) were selected. Moreover, papers reporting data for less than 10 compounds were excluded from the analysis. Furthermore, KIND contains the indication of the clinical phase the molecule has reached.

The K4DD consortium merged the efforts of 22 partners from European academia and the pharmaceutical industry in order to explore the role of kinetics in drug discovery. The kinetic data points collected were mainly derived from SPR experiments, radioligand binding assays, ITC and kPCA. The data collected were enriched with assay conditions like different buffers or duration of the experiments. All collected information was used to populate the database for the K4DD project. Upon the end of the project, all non-confidential data were transferred to ChEMBL^43^ (http://chembl.blogspot.com/2018/05/chembl-24-released.html), and all data are available following the ChEMBL document ID CHEMBL3885741.

**Fig. 1:**
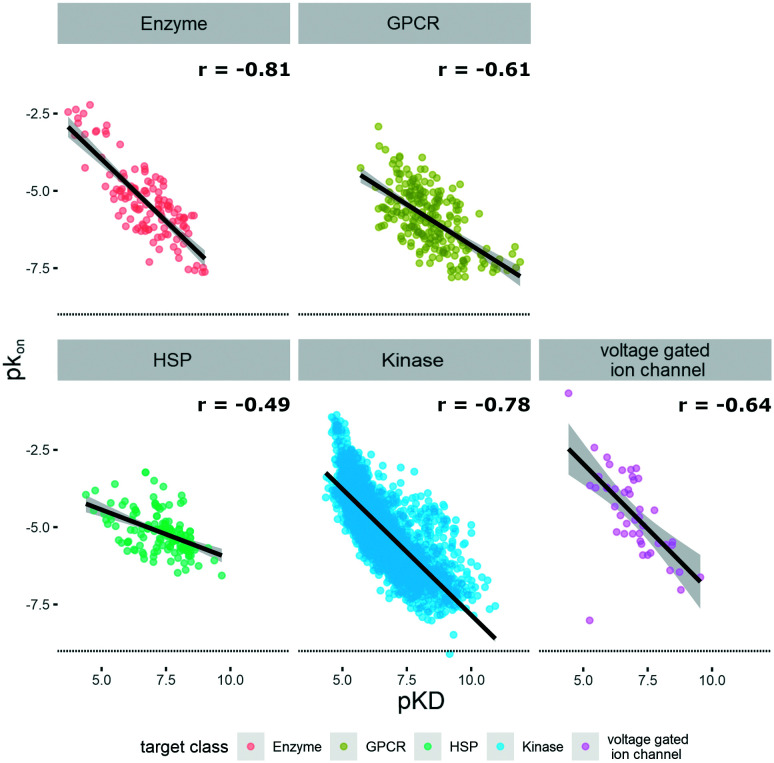
Correlation of affinity and on-rates, where p*K*_D_ is plotted on the *x*-axis and p*k*_on_ is plotted on the *y*-axis. Target families are displayed in different colours. Regression line is indicated, and error bars are shaded in grey. Pearson's *R* coefficient (*r*) for each class is displayed. Further details are reported in Table S1.† R 3.6.3 was used for statistical analysis and visualization.

**Fig. 2:**
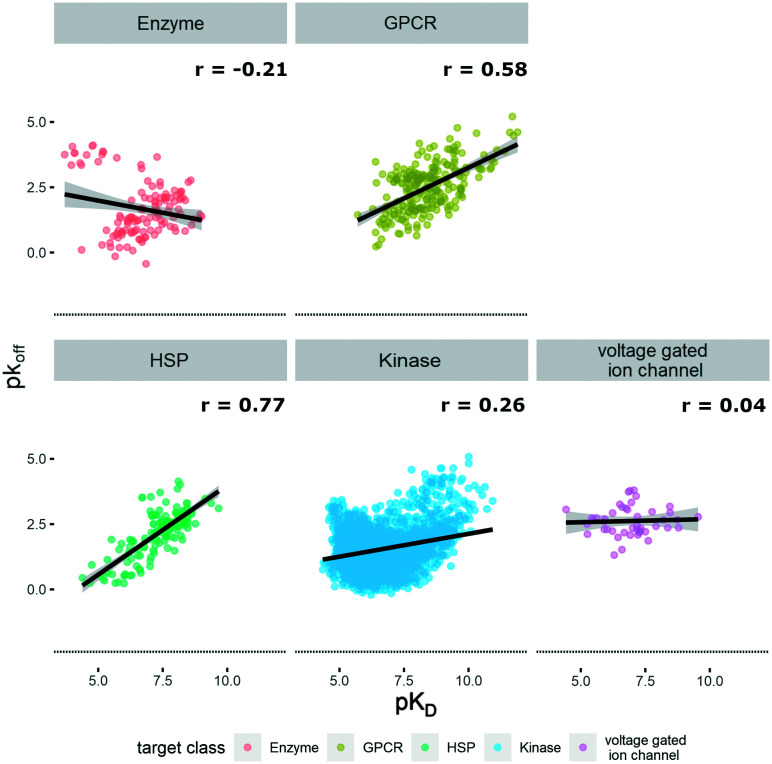
Correlation of affinity and off-rates, where p*K*_D_ is plotted on the *x*-axis and p*k*_off_ is plotted on the *y*-axis. Target families are displayed in different colours. Regression line is indicated, and error bars are shaded in grey. Pearson's *R* coefficient (*r*) for each class is displayed. Further details are reported in Table S2.† R 3.6.3 was used for statistical analysis and visualization.

The KIND dataset contains 78 biological targets, comprising 3238 data triplets for kinases, 242 for GPCRs, 160 for heat shock proteins (HSPs), 127 for enzymes, and 45 for ion channels. To give a general overview on the distribution of physicochemical properties, three relevant ones were chosen to examine the dataset's property distribution. The three descriptors mentioned are log *P*(o/w), TPSA, and molecular weight, and the respective graphs for the different target classes of the database can be found in Fig. S1.† The log *P*(o/w) was chosen as a measure of hydrophobicity of a compound, while the TPSA was chosen to represent polarity. This large dataset offered the opportunity to analyze and extrapolate general trends of kinetic behavior of compounds on different targets. The analysis was limited to the available data, which in the case of the ion channels was a single publication reporting kinetic data of hERG inhibitors.^31^ In this case all the compounds display rather high lipophilicity values, which is a relevant property for hERG inhibition and explains the shift of the property distribution in Fig. S1.† The correlations of on-rates (displayed as p*k*_on_) and affinity (p*K*_D_) for different target classes (Fig. 1) indicate that for most target classes, the on-rates and corresponding affinity values show a negative correlation. The same trend across target classes cannot be observed for the correlation between off-rates (displayed as p*k*_off_) and affinity (p*K*_D_) (Fig. 2).

Thus, the effect where ameliorated affinity accelerates binding seems to be a general trend. However, elaborating on specific examples (see the Case studies section) showcases opportunities on how on-kinetics can be influenced independently from affinity.

### Matched molecular pair (MMP) dataset and its analysis

In order to elucidate the impact small structural changes might have on the kinetic behavior of a molecule, we analyzed in total 395 matched molecular pairs (MMPs) extracted from KIND. Such pairs are composed of two molecules possessing an identical scaffold and showing one minor chemical modification (*i.e.* introduction of a substituent onto an unsubstituted aromatic ring, or replacement of a functional group by another group).

**Fig. 3:**
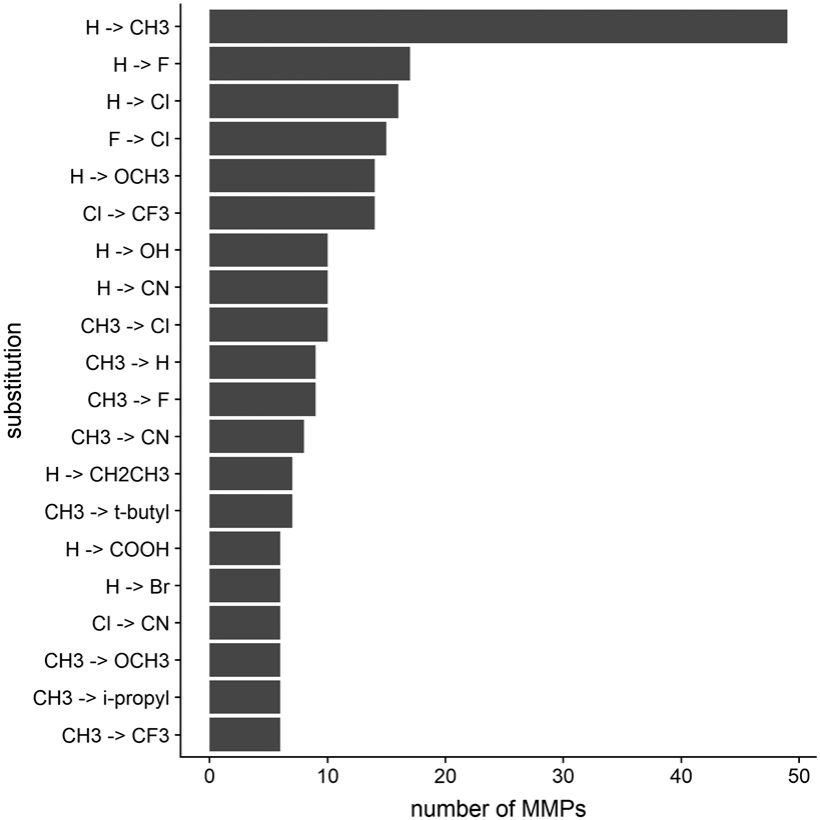
The 20 most common substitutions among the MMPs of the dataset are depicted. Substitutions are reported according to the molecules' increase in polarity (calculated as the overall increase of TPSA). R 3.6.3 was used for statistical analysis and visualization.

The dataset includes a variety of different modifications. The top 20 modifications represent less than 65% of the entire dataset, while the most common modification, which is the introduction of a methyl group to replace a hydrogen atom, comprises around 15%. The 20 most common transformations found in the MMP dataset are reported in Fig. 3. These chemical substitutions are in fact moieties which are prominently used in a medicinal chemistry context to optimize compounds in the drug discovery pipeline.

**Fig. 4:**
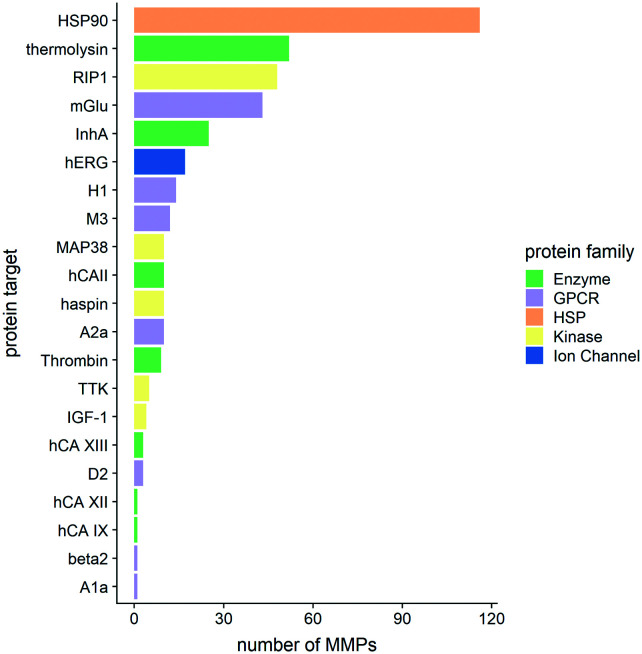
The numbers of matched molecular pairs (MMPs) per protein target are shown. Colour codes refer to the protein family a target belongs to. R 3.6.3 was used for statistical analysis and visualization.

Fig. 4 shows the distribution of the MMPs among different protein targets and how they cluster in various protein families.

We previously demonstrated that changes in a molecule's polarity are the major factor for *k*_on_ alteration in Hsp90.^24^ In order to investigate whether this hypothesis can be generalized across targets, we analyzed KIND by focusing on the MMPs with the highest differences in *k*_on_ values. All pairs were sorted according to decreasing *k*_on_, and the top 20 were selected for further analysis (Table 1).

**Table 1:** 20 MMPs with the highest values of Δp*k*_on_ (slowdown in the association rate due to the chemical substitution). Kinetic parameters Δp*k*_off_ and Δp*K*_D_ as well as biological (target and target class) and chemical data (MMP summary and ΔTPSA) are included

Δp*k*_on_	Δp*k*_off_	Δp*K*_D_	MMP_summary	Target	Target_class	ΔTPSA
2.06	−0.13	−2.15	CH_3_ → COOH	hERG	Voltage gated ion channel	37.30
1.98	−0.24	−2.22	H → CH(CH_3_)NHCH_3_	MAP38	Kinase	3.24
1.71	1.44	−0.27	CH_3_ → H	H1	GPCR	11.00
1.64	−0.09	−1.73	CH_3_ → H	H1	GPCR	11.00
1.59	1.00	−0.60	H → CH_2_COOH	H1	GPCR	37.30
1.56	2.82	1.26	C(=O)OCH_3_ → COOH	H1	GPCR	11.00
1.51	−0.19	−1.68	CH_3_ → CN	hERG	Voltage gated ion channel	23.79
1.50	0.37	−1.39	H → COOH	hERG	Voltage gated ion channel	37.30
1.48	1.29	−0.20	C(=O)OCH_3_ → COOH	H1	GPCR	11.00
1.44	−0.02	−1.05	CH_2_CH_3_ → H	hERG	Voltage gated ion channel	8.79
1.39	0.05	−1.34	Br → COOH	HSP90	HSP	37.30
1.34	0.30	−1.02	CH_3_ → *t*-butyl	HSP90	HSP	0.00
1.19	−0.06	−1.19	H → F	hERG	Voltage gated ion channel	0.00
1.19	0.59	−0.60	OCH_3_ → COOH	H1	GPCR	17.07
1.16	0.02	−1.14	CH_3_ → F	HSP90	HSP	0.00
1.11	0.49	−0.67	H → OCH_3_	HSP90	HSP	9.23
1.11	0.10	−1.01	CH_3_ → F	TTK	Kinase	0.00
1.11	0.13	−0.98	H → OCH_2_CH_3_	HSP90	HSP	9.23
1.04	1.97	0.94	OCH_3_ → COOH	H1	GPCR	17.07
1.03	−0.66	−1.69	H → CH(CH_3_)NHCH_3_	MAP38	Kinase	3.24

All five different protein families are present in the top 20 positions, granting diversity of the subset. For nearly all the MMPs (16 out of 20) a substitution that increases polarity is reported. This is a general finding which can be observed across the entire dataset. The largest differences in on-rates were found when introducing charged moieties. By introducing those moieties, a slowdown of the on-rate of 0.5 up to 2 orders of magnitude could be observed. The responsible for such a *k*_on_ decrease might be: i) the electrostatic repulsion (*e.g.* a charged moiety that transits through a binding pathway which displays similar electrostatic characteristics) and/or ii) desolvation penalties (*e.g.* a polar moiety that traverses through a hydrophobic passage and therefore needs to strip off all water molecules solvating it). Among the 20 pairs examined, 18 are accompanied by a concomitant impairment of affinity. This is expected if a modification on the ligand doesn't provide any additional interaction once the molecule accommodates its bound pose within the binding site. Conversely, if such modifications establish additional interactions in the final bound complex, an improvement in affinity can be achieved. The latter could be observed in two of our proposed case studies (Case study 1 and Case study 3 discussed in detail below). A concurrent slowdown of the on-rate and improvement of *K*_D_ results in a prolonged residence time.

Apart from the association rate constant *k*_on_, we also analyzed the dissociation rate constant *k*_off_. Following the procedure we established for the on-rate, we sorted the MMP dataset according to the biggest difference in *k*_off_, and the 20 pairs showing the most pronounced difference in dissociation rates were selected (Table 2).

**Table 2:** 20 MMPs displaying the highest values of Δp*k*_off_ (slowdown in the dissociation rate resulting from chemical modification). Kinetic parameters Δp*k*_on_ and Δp*K*_D_ as well as biological (target and target class) and chemical data (MMP summary and ΔTPSA) are included

Δp*k*_off_	Δp*k*_on_	Δp*K*_D_	MMP_summary	Target	Target_class	ΔTPSA
2.82	1.56	1.26	C(=O)OCH_3_ → COOH	H1	GPCR	11.00
2.44	−1.68	3.99	H → I	Haspin	Kinase	0.00
2.23	−1.03	3.20	H → OH	A2a	GPCR	20.23
2.14	0.06	1.80	CH_3_ → H	M3	GPCR	11.00
2.09	0.89	1.20	H → COOH	Thermolysin	Enzyme	40.13
2.02	−0.66	2.42	F → I	Haspin	Kinase	0.00
1.97	1.04	0.94	OCH_3_ → COOH	H1	GPCR	17.07
1.79	0.32	1.48	H → COOH	Thermolysin	Enzyme	40.13
1.75	0.73	0.70	H → OH	M3	GPCR	20.23
1.74	−1.69	3.35	H → Br	Haspin	Kinase	0.00
1.66	−1.15	2.77	H → Cl	Haspin	Kinase	0.00
1.56	0.08	1.40	OH → CH_2_OH	M3	GPCR	0.00
1.44	1.71	−0.27	CH_3_ → H	H1	GPCR	11.00
1.40	−0.38	1.78	H → CH_3_	MAP38	Kinase	0.00
1.34	0.71	0.61	H → OCH_3_	IGF-1	Kinase	9.23
1.33	0.76	0.30	CH_3_ → OH	M3	GPCR	20.23
1.32	−0.67	1.79	F → Br	Haspin	Kinase	0.00
1.29	1.48	−0.20	C(=O)OCH_3_ → COOH	H1	GPCR	11.00
1.23	−0.13	1.21	F → Cl	Haspin	Kinase	0.00
1.21	−0.19	1.40	F → CN	IGF-1	Kinase	12.03

**Fig. 5:**
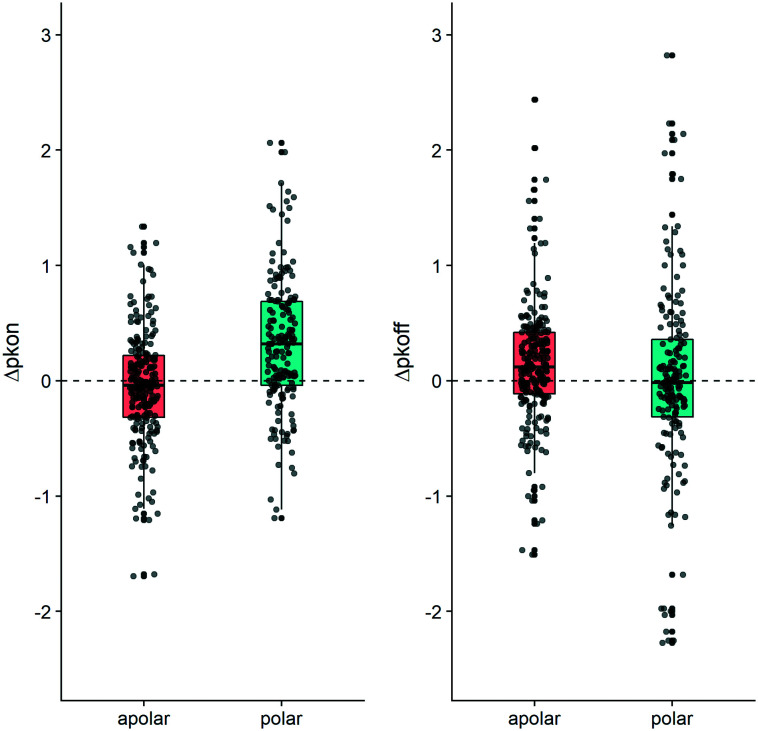
Boxplot depicting the contribution to Δp*k*_on_ (left-hand side) and to Δp*k*_off_ (right-hand side). The MMP dataset has been divided according to the shift in ΔTPSA. The “apolar” boxplot (coral color) exemplifies matched pairs which do not show a change in TPSA. The “polar” (dark cyan color) boxplot depicts matched molecular pairs for which the TPSA value changed (*p*-values reported in Table S3†). R 3.6.3 was used for statistical analysis and visualization.

Conversely to the *k*_on_ data, a change in polarity in the MMPs did not produce a consistent shift in the average value for *k*_off_. The plots in Fig. 5 illustrate that the behavior we observed for the 20 examined MMPs can be seen for the entire dataset. Fig. 5 furthermore exemplifies how polar substitutions affect on-rates significantly differently from apolar substitutions (Wilcoxon signed rank test *p*-value = 1.62 × 10^−10^ and *p*-value = 0.16 respectively), with almost 75% of the data points showing an increase of Δp*k*_on_ (polar box-plot in dark cyan, on the left-hand side in Fig. 5). However, an analogous impact of the polarity variation on Δp*k*_off_ cannot be retrieved (polar box-plot in dark cyan on the right-hand side of Fig. 5).

**Fig. 6:**
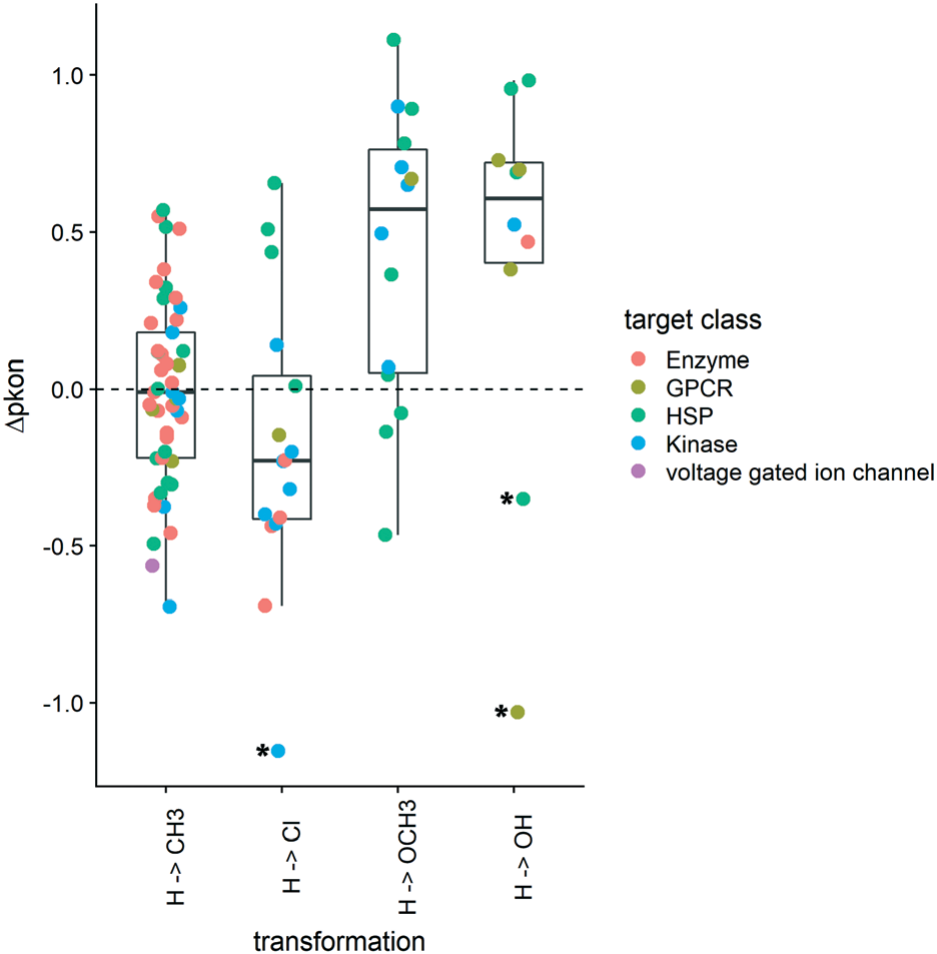
Boxplots of Δp*k*_on_ for matched molecular pair analysis. The substitution pattern is shown on the *x*-axis and the change in p*k*_on_ (Δp*k*_on_) is depicted on the *y*-axis. Left to right: the first two boxplots show substitution patterns toward decreased polarity, while boxplots three and four showcase compound pairs with increased polarity. The respective target classes are represented using color codes. Asterisks mark outliers. R 3.6.3 was used for statistical analysis and visualization.

As the distribution of *k*_on_ values varies according to target classes, we are looking at the change of the on-rates rather than absolute values. These Δp*k*_on_ values showcase how a change of substitution affects the on-rate in a positive or a negative way. An increase in p*k*_on_ (+Δp*k*_on_) leads to a slower on-rate, while a decrease in p*k*_on_ (−Δp*k*_on_) speeds up the binding of the small molecule to its protein target. The boxplots in Fig. 6 depict a set of specific cases of a hydrogen atom being substituted by CH_3_, Cl, OCH_3_, or OH. Although exchange by a methyl group leads to a variety of effects on *k*_on_ (including the increase and decrease of on-rates), the mean change is close to 0. Overall, for this MMP no general trend can be deduced across target classes, or even within one target class. In contrast, substitution of H by a methoxy group leads to a slowdown of molecules acting on kinases and most HSPs as well as the example we could obtain for GPCR ligands. Alike, the collected examples for hydroxylated compounds show a similar slowdown in on-rates.

**Fig. 7:**
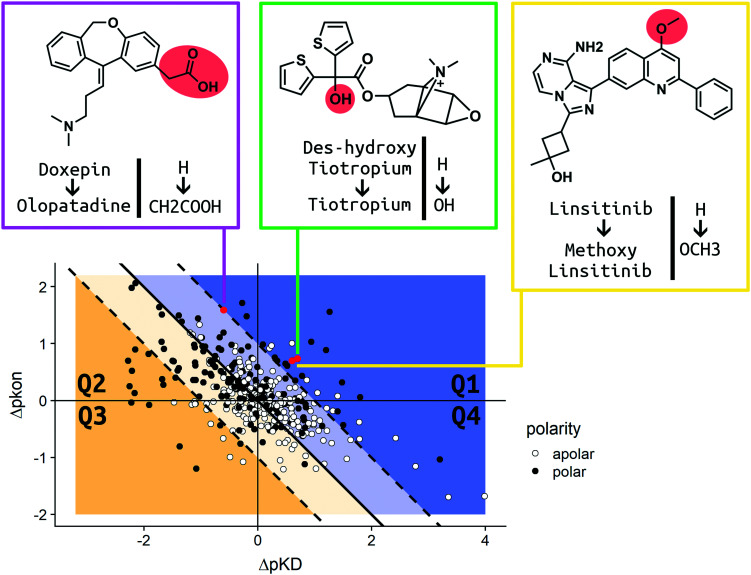
The kinetic map shows the correlation of the variation of p*k*_on_ and p*K*_D_ for the MMPs of the dataset. The map is divided into four quadrants: Q1–Q4. The blue area hosts MMPs for which the residence time has increased. The yellow area comprises points exhibiting decreased residence times. The Δp*k*_off_ value is encoded by the perpendicular distance from the Q2–Q4 bisecting line. White dots illustrate substitution patterns with unaltered TPSA values (42 in Q1, 56 in Q2, 87 in Q3 and 37 in Q4), while black dots represent substitution patterns with changed TPSA values (27 in Q1, 97 in Q2, 29 in Q3 and 20 in Q4). Red points highlight the MMPs discussed in further detail in the Case studies section of the Results and discussion. Olopatadine (pink box), tiotropium (green box) and linsitinib (yellow box) are depicted in 2D. R 3.6.3 was used for statistical analysis and visualization. Chemical structures were drawn using ChemDraw 14.

In order to present an overview on the results in a visual manner, we constructed a kinetic map for the MMPs (Fig. 7). The map describes the MMPs according to their shift in p*K*_D_ and p*k*_on_ (*x* and *y* axis, respectively) with additional information on the respective change in the TPSA profile. Due to the relationship of Δp*K*_D_ and Δp*k*_on_, (*K*_D_ = *k*_off_/*k*_on_) it is also possible to visualize Δp*k*_off_ (diagonal lines) on the same chart. White dots are used to report MMPs for which no difference in the calculated TPSA was observed. Black dots show all pairs whose polarity was impacted due to the introduced ligand modification (for consistency, all the pair transformations are written to display an increase in ΔTPSA for the MMP). The map has been divided into four quadrants. In Q1 (top right corner) the MMPs are reported, which show an increase in residence time by both stabilizing the bound state (amelioration of p*K*_D_) and destabilizing the transition state (slowdown of p*k*_on_) after substitution.

The modifications observed in Q1 constitute the best-case scenario in terms of prolonging the residence time inasmuch as the change produces a ligand with longer binding (increased Δp*k*_off_). Q2 (top left corner) includes those pairs which show a destabilization in their transition state (positive Δp*k*_on_ therefore, a slowdown of the on-rate), but a simultaneous reduction in affinity (negative Δp*K*_D_ therefore, a loss in affinity). Due to the large variation, we observed cases in which the alterations produced molecules with an increased residence time (blue Q2 area) as well as a reduced residence time (yellow Q2 area). The Q3 quadrant (bottom left corner) covers MMPs whose alteration resulted in a decrease of affinity (negative Δp*K*_D_) and an increase of the on-rate (negative Δp*k*_on_). The residence time is decreased for all pairs found in Q3. Q4 (bottom right quadrant) contains matched pair values which are derived from chemical modifications which increase affinity (positive Δp*K*_D_) and trigger faster binding (negative Δp*k*_on_). Similar to Q2, the variation of the Δp*k*_off_ for this quadrant depends on the shift of Δp*k*_on_ and Δp*K*_D_. All cases resulting in a prolonged residence time are placed in the blue area of Q4. The yellow area comprises MMPs for which the ameliorated p*K*_D_ didn't compensate the faster binding, which results in a decreased residence time. For a more detailed analysis, we chose three relevant MMPs to discuss their kinetic parameter shifts in the Case studies section.

### Case studies

In order to discuss the trends observed in more detail, we present three case studies, which were chosen according to the different scenarios reported and visualized in our analysis (Fig. 7). As we aim to impact drug–target kinetics toward prolonged residence time benefiting from transition state destabilization, regardless of the change in affinity, we chose examples from quadrants Q1 and Q2. Tiotropium and linsitinib, as well as their matched pair analogues, represent the ideal scenario for a lead optimization program that aims to find molecules with high affinity and long residence time.

### Case study 1 (Q1; H–OH)

**Fig. 8:**
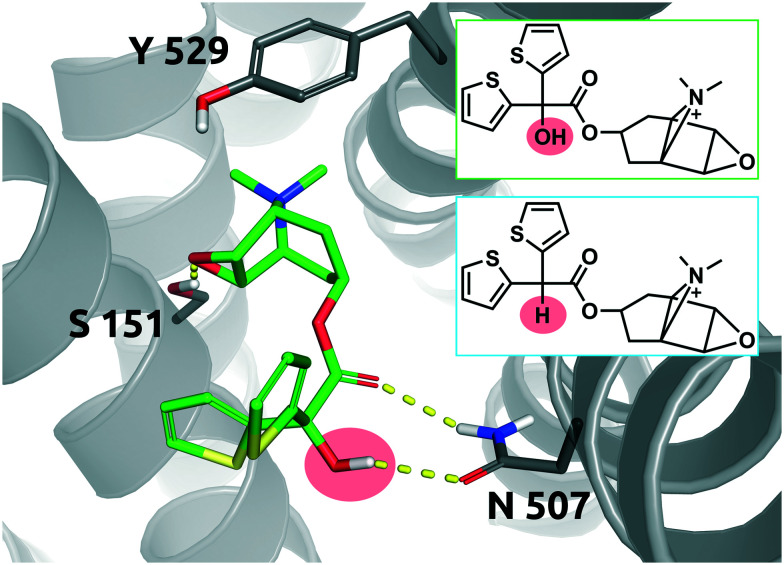
2D representation of the small molecule tiotropium (green box). Tiotropium bound to the binding pocket of the M3 muscarinic acetylcholine receptor (PDB: 4DAJ). Tiotropium is shown in green, stick representation. The hydroxyl group performing the polar interaction inside the binding pocket is highlighted in red. Des-hydroxy tiotropium derived from a study conducted by Tautermann *et al.*^30^ is depicted in 2D for comparison (cyan box). Residues of the M3 receptor are depicted in grey. Interactions of the drug and the protein are visualized in dashed yellow lines. PyMol 2.7 was used for visualization of the protein and small molecule. ChemDraw 14 was employed to show the 2D depiction.

The long-acting muscarinic antagonist tiotropium, which shows very high affinity for the M3 muscarinic acetylcholine receptor (p*K*_D_: 11.7, corresponding to 2 pM) remains bound to its receptor for 2.724 minutes.^30^Fig. 8 shows numerous interactions of the molecule with its protein target. The area highlighted in red indicates a hydrogen bond of the hydroxyl group on tiotropium and the carbonyl group on Asn507. The hydroxyl group acts as a H-bond donor. Its structural analogue, des-hydroxy tiotropium, is shown in the cyan box of Fig. 8. As the analogue lacks the hydroxyl group it is not expected to participate in the protein–ligand interaction the hydroxyl group was engaged in. The more apolar compound des-hydroxy tiotropium displays a faster on-rate, when compared to tiotropium, and a concomitant worsening of p*K*_D_ (p*K*_D_ des-hydroxy tiotropium: 11.0, corresponding to 10 pM). The energy barrier which des-hydroxy tiotropium has to overcome has been calculated to be 1.18 kcal mol^−1^ lower than the one of tiotropium.^30^

The 5-fold increase in affinity of tiotropium *vs.* des-hydroxy tiotropium and the accompanying 5.5-fold slowdown of the on-rate (*k*_on_) result in a 56-fold increase in residence time. This compound pair thus represents a good example for transformations in quadrant Q1 (Fig. 7).

### Case study 2 (Q2; H–CH_2_COOH)

**Fig. 9:**
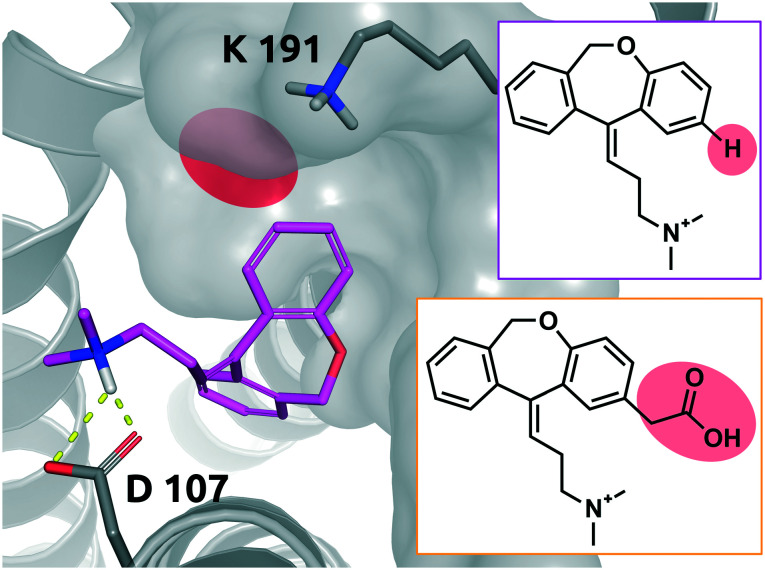
2D representation of the small molecule doxepin (magenta box). Doxepin bound to the binding pocket of the histamine H1 receptor (PDB: 3RZE). Doxepin is shown in magenta, stick representation. The substitution site that will accommodate the substituent for the matched molecular pair olopatadine is highlighted in red. Olopatadine derived from a study conducted by Shimamura *et al.*^45^ is depicted in 2D for comparison (orange box). Residues of the H1 receptor are depicted in grey. Interactions of the drug and the protein are visualized in dashed yellow lines. PyMol 2.7 was used for visualization of the protein and small molecule. ChemDraw 14 was employed to show the 2D depiction.

Doxepin is a tricyclic antidepressant with histamine H1 receptor antagonist properties. It inhibits H1, H2, 5-HT2A, 5-HT2B, muscarinic acetylcholine receptors M1–M5, alpha1 and alpha2 adrenergic receptors, and the D2 receptor.^44^ Bosma *et al.* reported that olopatadine, which is a selective histamine H1 antagonist, exhibits a 39-fold slower on-rate than doxepin. Fig. 9 shows doxepin in its bound pose (PDB: 3RZE) and olopatadine, which is expected to accommodate the binding pocket in a similar fashion.

The drugs show a less than 4-fold difference in *K*_D_ to the H1 receptor (doxepin: 0.8 nM, olopatadine: 3.1 nM); however, the residence time of doxepin is reported to be around 22 minutes, while olopatadine is determined to remain bound for 170 minutes.^46^ The affinity could not be fully preserved; however, a 39-fold prolongation of the on-rate and a 10-fold prolongation of the off-rate could be achieved by substitution of a hydrogen atom by carboxymethyl. The doxepin–olopatadine matched molecular pair showcases an example of a prolonged residence time mainly driven by an increase of Δp*k*_on_, passing from *k*_on_ of 1.17 × 10^6^ M^−1^ s^−1^ for doxepin to a value of 3 × 10^4^ M^−1^ s^−1^ for olopatadine. In fact, the destabilization of the transition state poses a significant barrier for the unbinding of the molecule from its bound state.

### Case study 3 (Q1; H–OCH_3_)

Linsitinib and its analogues are small molecules that inhibit the type 1 insulin-like growth factor (IGF-1) receptor, a well-known cell survival pathway activator and tumor growth promoter.^47^ PQIP, an analogue of linsitinib, has been crystallized in complex with its receptor. PQIP is structurally very similar to linsitinib and its methoxylated analogue.

**Fig. 10:**
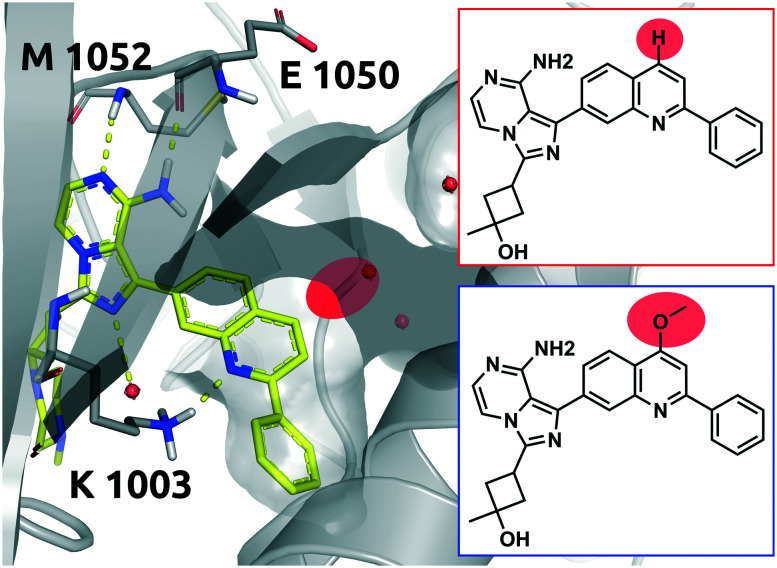
2D representation of the small molecule linsitinib (red box). As a reference the linsitinib analogue PQIP bound to the binding pocket of the insulin-like growth factor 1 (IGF-1) receptor (PDB: 3D94) is shown in yellow. PQIP^48^ and linsitinib are assumed to accommodate similar binding poses, inasmuch as their structural differences can be found on the solvent exposed side only. The red circle highlights the location of the matched molecular pair substitution. The methoxylated linsitinib derived from a study by Jin *et al.*^49^ is depicted in 2D for comparison (blue box). Residues of the IGF-1 receptor are depicted in grey. Interactions of the drug and the protein are visualized in dashed yellow lines. Water molecules are represented as red spheres. PyMol 2.7 was used for visualization of the protein and small molecule. ChemDraw 14 was employed to show the 2D depiction.

Therefore, PQIP has been used for our structural study, as a similar binding mode for linsitinib and its methoxylated analogue might be assumed. From SPR studies conducted within the K4DD consortium, linsitinib and methoxy-linsitinib show around 4-fold differences in *K*_D_ with the more polar compound being the more affine (linsitinib: 55.8 nM, methoxy-linsitinib: 13.8 nM). Such an increase in affinity might be explained by the introduction of a polar moiety in a fairly polar area, in which water molecules can be found if no ligand is bound. The crystal structure of PQIP bound to the IGF-1 receptor shows such water molecules in close vicinity of the substitution site of the MMP (Fig. 10). Moreover, the increase of the molecule's polarity generates a slower entrance (linsitinib: 8.92 × 10^4^ M^−1^ s^−1^, methoxy-linsitinib: 1.75 × 10^4^ M^−1^ s^−1^), resulting in a prolonged residence time for the methoxylated molecule with respect to the approved drug (linsitinib: 3.78 minutes, methoxy-linsitinib: 82.46 minutes). The introduction of a moiety that mildly disrupts the entry pathway by increasing the energy of the transition state, in combination with the favorable interactions once it reaches the bound state, locates this MMP in the Q1 quadrant of Fig. 7. Similar to Case study 1, the increase of the residence time is due to both the affinity increase and a slowdown of the access.

## Conclusions

### Thermodynamic and kinetic molecular basis

To the best of our knowledge, our dataset KIND is the largest publicly available kinetic dataset so far, comprising a total of 3812 small molecules. Taking advantage of the abundance of data, we could illustrate how to trigger the kinetic behavior of small molecules and derive more generalized trends. One of our key findings illustrates that *k*_on_ generally correlates better with *K*_D_ than *k*_off_ does (shown in Fig. 1 and 2). This trend can generally be observed among GPCRs, ion channels and soluble proteins.

In our work, we have provided examples for slowing down association rates by introducing polar moieties to a small molecule, which will have to be desolvated while entering the binding pocket.^24,50^ Furthermore, we have provided structural insight on how the residence time can remain unaltered, even though individual contributions of *K*_D_ and *k*_on_ change significantly. The trend we aim to illustrate is that the addition of polar moieties to small molecules tends to affect on-rates if their desolvation is part of the binding process. Those trends, which were showcased for proteins affiliated with three different families, could be extrapolated to other protein families and provide a more generalized scheme to trigger kinetic parameters, specifically for a hydrophobic pocket environment. Various MMPs presented in this work impact the association rate significantly, and some of them result in altered residence times. This could be achieved due to the introduction of an increased energy barrier along the (un)binding pathway (kinetic contribution) and furthermore, by additionally established interactions of the aforementioned polar groups once the molecule is bound (thermodynamic contribution). Putting our findings in context with scientific publications on enthalpic and entropic contributions to on- and off- rates (Fig. S2 and Table S4†), the enthalpic signature of the on-rate, which contributes to the energy barrier, is predominant.

The gained knowledge about how to trigger kinetic parameters of small molecules binding to protein targets is valuable information. For different targets diverse ranges of residence times are considered to be optimal. Therefore, the ability to tailor a compound's residence time according to its biological target and the desired effect would be the best-case scenario.

In future analysis, we want to extend our studies to include information about the protein binding pocket environment using the matched pair (MMP) analysis. Employing grid based methods^48^ will allow us to distinguish between different binding pockets. This more complete perspective might identify the preconditions for different kinds of substituents to achieve both the slowdown of the binding event (introduction of an energy barrier along the pathway) and simultaneous stabilization of the ground state (improving affinity for the receptor) on a broader scale. Optimization of binding kinetics of course is a complex process with many factors contributing. Nevertheless, with this contribution we aimed to shed light on this still underexplored field by providing guidance for a more rationalized modification of molecules in order to effectively steer the residence time in the context of lead optimization.

## Conflicts of interest

Gerhard F. Ecker is co-founder of Phenaris GmbH.

## Supplementary Material

MD-011-D0MD00178C-s001

MD-011-D0MD00178C-s002
