# Supplementary material for: A structure–kinetic relationship study using matched molecular pair analysis
Source: RSC Med Chem. 2020 Sep 21;11(11):1285–94. doi: 10.1039/d0md00178c (PMC8126976; doi:10.1039/d0md00178c)
Supplement: MD-011-D0MD00178C-s002 [file MD-011-D0MD00178C-s002.pdf]

Electronic Supplementary Material (ESI) for RSC Medicinal Chemistry.
This journal is © The Royal Society of Chemistry 2020

Supplementary Information

A structure-kinetic relationship study using Matched Molecular Pair analysis.

Doris A. Schuetz[1,‡], Lars Richter[1,‡], Riccardo Martini[1,‡], Gerhard F. Ecker[1,]*

[1] University of Vienna, Department of Pharmaceutical Chemistry, UZA 2, Althanstrasse 14,

1090 Vienna, Austria

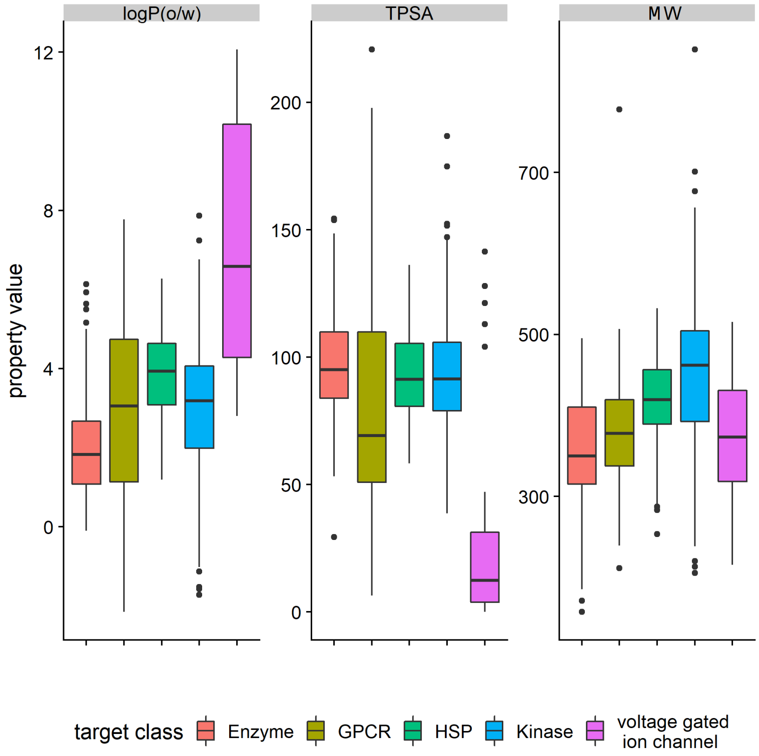

Figure S1. Distribution of the properties “logP(o/w)”, “TPSA” (topological polar surface area) and “MW” (Molecular Weight) for various target classes (color coded). Whiskers indicate the distribution range of all data points; black dots illustrate outliers. The “property values” were calculated for each compound in the dataset, using Molecular Operating Environment Software (MOE, Version 2018.01. Chemical Computing Group Inc., Montreal Canada). R 3.6.3. was used for statistical analysis and visualization.

Table S1. Correlation details of pK_D and pk_{on}, calculated for five different target families. For each of them the Pearson's R coefficient (r) together with the lower and upper interval confidence 95% (95% CI) are reported.

Target family	Pearson's R (r)	Lower Confidence Interval (95% CI)	Upper Confidence Interval (95% CI)
Enzyme	-0.81	-0.86	-0.74
GPCR	-0.61	-0.69	-0.53
HSP	-0.49	-0.60	-0.37
Kinase	-0.78	-0.80	-0.77
Voltage gated ion channel	-0.64	-0.79	-0.43

Table S2 Correlation details of pK_D and pk_{off}, calculated for five different target families. For each of them the Pearson's R coefficient (r) together with the lower and upper interval confidence 95% (95% CI) are reported.

Target family	Pearson's R (r)	Lower Confidence Interval (95% CI)	Upper Confidence Interval (95% CI)
Enzyme	-0.21	-0.37	-0.04
GPCR	0.58	0.49	0.66
HSP	0.77	0.70	0.83
Kinase	0.26	0.23	0.30
Voltage gated ion channel	0.04	-0.25	0.33

Table S3. One-sample Wilcoxon signed rank test details of polar and apolar substitutions for Δpk_{on} and Δpk_{off} to assess their deviation from 0. For each data series, the p-value is reported.

Data Series	p-value
Apolar-Δpk_{on}	0.1599
Polar - Δpk_{on}	$1.621e^{-10}$
Apolar - Δpk_{off}	$8e^{-6}$
Polar - Δpk_{off}	0.8936

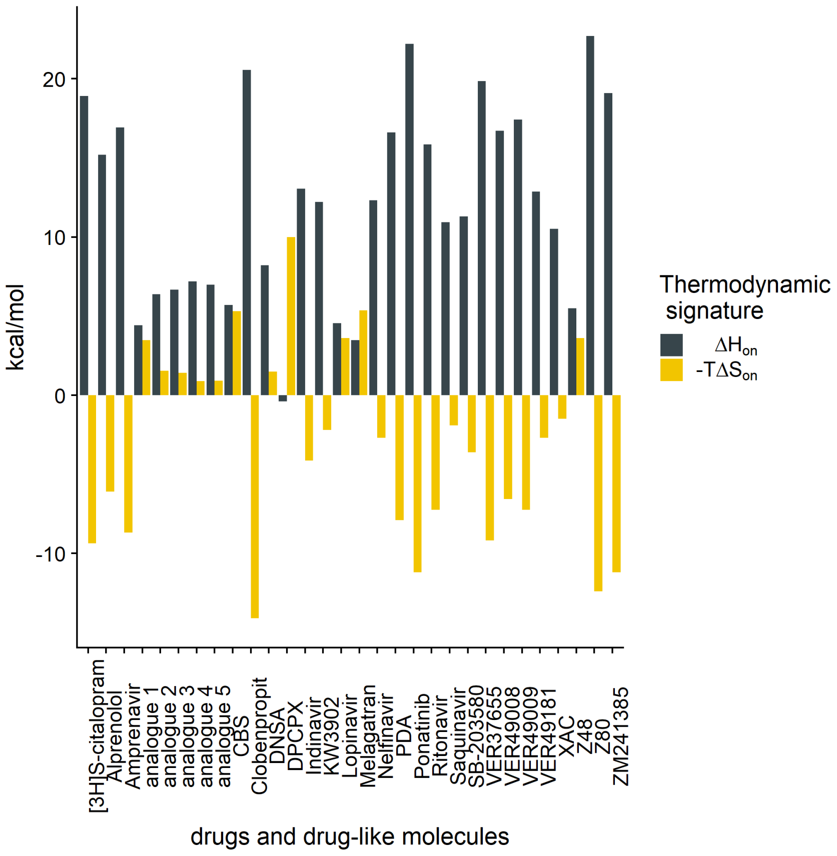

Figure S2. Measured thermodynamic signature of drug-like molecules
Enthalpic and entropic contribution of drugs and drug-like molecules to on- and off-rates. Drugs and drug-like molecules are plotted on the x-axis, while Energy changes in kcal/mol are indicated on the y-axis. A penalty in terms of enthalpy can be detected for the majority of the reported drugs.

Table S4. Information on Figure S1.

Target	Protein class	Ligand	dG_{on}	dH_{on}	$mTdS_{on}$	dG	dH	mTdS	Reference
gpH3 - receptor	GPCR	Clobenpropit	6.45	20.55	-14.1	-11.7	-4.1	-7.6	[1]
b2 adrenoceptor	GPCR	Alprenolol	9.2	15.2	-6.1	-12.3	-3.5	-8.8	[2]
A2a receptor	GPCR	ZM241385	7.9	19.1	-11.2	-12.9	-21.5	8.6	[3]
A2a receptor	GPCR	XAC	9	10.5	-1.5	-10.1	-6.2	-3.9	[3]
A2a receptor	GPCR	DPCPX	9.6	-0.4	10	-9.8	-8.8	-1	[3]
A2a receptor	GPCR	KW3902	10	12.2	-2.2	-8.9	1	-9.9	[3]
A2a receptor	GPCR	Z80	10.3	22.7	-12.4	-7.9	3.1	-11	[3]
A2a receptor	GPCR	Z48	9.1	5.5	3.6	-9.7	-11	1.3	[3]
HIV-1 protease	Protease	Amprenavir	8.24	16.93	-8.69	-12.5	3.7	-16.1	[4]
HIV-1 protease	Protease	Indinavir	8.92	13.04	-4.12	-12	5.4	-17.4	[4]
HIV-1 protease	Protease	Lopinavir	8.15	4.54	3.61	-13.6	2.2	-15.9	[4]
HIV-1 protease	Protease	Nelfinavir	9.62	12.31	-2.69	-11.5	5.4	-16.9	[4]
HIV-1 protease	Protease	Ritonavir	8.61	15.86	-7.25	-12.4	7	-19.4	[4]
HIV-1 protease	Protease	Saquinavir	9.04	10.94	-1.9	-13	2.7	-15.7	[4]
Thrombin	Protease	Melagatran	8.86	3.49	5.37	-11.2	-6.6	-4.6	[5]
Thrombin	Protease	analogue 1	7.9	4.42	3.48	-11.3	-5.1	-6.2	[5]
Thrombin	Protease	analogue 2	7.91	6.37	1.55	-11.6	-5.5	-6.1	[5]
Thrombin	Protease	analogue 3	8.06	6.66	1.4	-12.1	-5.5	-6.6	[5]
Thrombin	Protease	analogue 4	8.08	7.18	0.9	-12	-5.8	-6.2	[5]

Thrombin	Protease	analogue 5	7.9	6.99	0.91	-12.1	-5.6	-6.6	[5]
HSP90	Kinase	VER37655	10.79	19.86	-9.17	-9	-1.5	-7.5	[6]
HSP90	Kinase	VER49181	10.08	12.86	-2.68	-10.3	-2.7	-7.6	[6]
HSP90	Kinase	VER49008	10.17	16.71	-6.55	-10.4	-3.8	-6.4	[6]
HSP90	Kinase	VER49009	10.16	17.41	-7.24	-10.4	-3.7	-6.7	[6]
FGFR1	Kinase	PDA	8.7	16.6	-7.9	-11	-12.1	1.1	[7]
FGFR1	Kinase	Ponatinib	11	22.2	-11.2	-10.8	-8.2	-2.6	[7]
Map38alpha	Kinase	SB-203580	7.7	11.3	-3.6	-10.7	-11.4	0.7	[8]
Serotonine Transporter	Transporter	[3H]S-citalopram	9.53	18.9	-9.37	-11.79	-6	-5.74	[9]
Carboanhydrase II	Enzyme	CBS	11	5.7	5.3	-8.3	-11.6	3.3	[10]
Carboanhydrase II	Enzyme	DNSA	9.7	8.2	1.5	-8.8	-5.7	-3.1	[10]

Supplementary Methods:

We employed PubMed as a search engine for extracting the kinetic triplets from the literatur, using the following key:
("residence time" OR "binding kinetics" OR "dissociation rate" OR "association rate") AND (koff OR kon)
The online resources were last accessed in January 2019. Only papers containing the numeric values for all three parameters investigated (K_D, k_{on} and k_{off}) were selected. Moreover, papers reporting data for less than 10 compounds were excluded from the analysis.

The dataset containing the raw data was filtered to exclude entries that reported “<” or “>” in their published relation field. Moreover, we converted KD, kon and koff in nM, M-1 s-1 and s-1 respectively. If a compound was measured multiple times in the same assay, we considered the average. Finally, the KD, kon and koff values are replaced by pKD, pkon and pkoff. As our focus was small molecules, peptide like molecules were excluded.

References:

1 A. Strasser and H. Joachim Wittmann, *J Phys Chem Biophys*, , DOI:10.4172/2161-0398.S1-001.
2 R. O. Dror, A. C. Pan, D. H. Arlow, D. W. Borhani, P. Maragakis, Y. Shan, H. Xu and D. E. Shaw, *PNAS*, 2011, **108**, 13118–13123.
3 G. Deganutti, A. Zhukov, F. Deflorian, S. Federico, G. Spalluto, R. M. Cooke, S. Moro, J. S. Mason and A. Bortolato, *In Silico Pharmacol.*, 2017, **5**, 16.
4 C. F. Shuman, M. D. Hämäläinen and U. H. Danielson, *J. Mol. Recognit.*, 2004, **17**, 106–119.
5 J. Winquist, S. Geschwindner, Y. Xue, L. Gustavsson, D. Musil, J. Deinum and U. H. Danielson, *Biochemistry*, 2013, **52**, 613–626.
6 P. Schmidtke, F. J. Luque, J. B. Murray and X. Barril, *J. Am. Chem. Soc.*, 2011, **133**, 18903–18910.
7 T. Klein, N. Vajpai, J. J. Phillips, G. Davies, G. A. Holdgate, C. Phillips, J. A. Tucker, R. A. Norman, A. D. Scott, D. R. Higazi, D. Lowe, G. S. Thompson and A. L. Breeze, *Nat Commun*, 2015, **6**, 7877.
8 D. Casper, M. Bukhtiyarova and E. B. Springman, *Analytical Biochemistry*, 2004, **325**, 126–136.
9 R. S. Martin, R. A. Henningsen, A. Suen, S. Apparsundaram, B. Leung, Z. Jia, R. K. Kondru and M. E. Milla, *J Pharmacol Exp Ther*, , DOI:10.1124/jpet.108.142307.
10 Y. S. N. Day, C. L. Baird, R. L. Rich and D. G. Myszka, *Protein Science*, 2002, **11**, 1017–1025.
